# Productive, Physiological, and Soil Microbiological Responses to Severe Water Stress During Fruit Maturity in a Super High-Density European Plum Orchard

**DOI:** 10.3390/plants14081222

**Published:** 2025-04-16

**Authors:** Arturo Calderón-Orellana, Gonzalo Plaza-Rojas, Macarena Gerding, Gabriela Huepe, Mathias Kuschel-Otárola, Richard M. Bastías, Tamara Alvear, Andrés Olivos, Mauricio Calderón-Orellana

**Affiliations:** 1 Departamento de Producción Vegetal, Facultad de Agronomía, Universidad de Concepción, Chillán 3780000, Chile; gplaza2017@udec.cl (G.P.-R.); mgerding@udec.cl (M.G.); ghuepe2017@udec.cl (G.H.); ribastias@udec.cl (R.M.B.); talvear2019@udec.cl (T.A.); 2 Departamento de Suelos y Recursos Naturales, Facultad de Agronomía, Universidad de Concepción, Chillán 3780000, Chile; mkuschel@udec.cl; 3 OLIVOS Riego Spa, Curicó 3340000, Chile; andres@olivos.cl (A.O.); mcalderon@olivos.cl (M.C.-O.)

**Keywords:** water productivity, late deficit irrigation, water stress, plant growth-promoting bacteria, fruit quality, prune orchard

## Abstract

The super high-density (SHD) production system has recently been introduced to the Chilean European plum (*Prunus domestica* L.) industry, but the potential of applying regulated deficit irrigation (RDI) in this system remains unexplored. As irrigation water availability in Chile has been strongly jeopardized by climate change, there is an urgent need to validate water-conserving practices in modern production systems. A field study was conducted in a commercial SHD European plum orchard (cv. French grafted on Rootpac-20 rootstock) for two consecutive seasons in Peralillo, O’Higgins Region, Chile. The objective of this study was to assess the impact of a late water deficit (LD) on water productivity, fruit quality, plant water relations, and soil microbiota. The results showed that implementing LD enhanced water productivity by 40% without compromising fresh and dry fruit quality. Moderate to severe water stress induced no changes in physiological parameters such as stomatal conductance and photochemical efficiency. Additionally, the LD treatment significantly reduced soil moisture but increased the abundance of certain groups of beneficial soil microbiota and fine roots. These results highlight the potential of LD as a viable water-conserving practice in modern SHD European plum orchards, particularly in regions facing water scarcity due to climate change.

## 1. Introduction

Chile is the world’s leading exporter of dried plums (prunes), with an annual production of nearly 100,000 tons [1,2]. The cultivar ‘D’Agen’ or ‘French’ constitutes over 95% of the 12,530 hectares planted with European plum trees (*Prunus domestica* L.) in Chile [3]. The conventional approach to cultivating this fruit crop includes establishing low plant densities, ranging from 400 to 500 plants per hectare, employing medium vigor rootstocks, such as Marianna 2624, Mirobalan, or Nemaguard, and using old training systems, such as the Italian vase or the central axis [4,5]. In general, mature European plum orchards require substantial irrigation during the growing season to achieve high yields (between 600 and 800 mm year^−1^) [6], as conventional management results in the cultivation of tall and vigorous trees (surpassing 5 m in height) capable of producing over 5000 fruits per plant. Yet, European plum trees are considered highly tolerant to severe water stress [7], which has facilitated the adoption of conservative irrigation strategies (e.g., regulated or sustained deficit irrigation) to reduce water application in areas affected by frequent and prolonged droughts.

Regulated deficit irrigation (RDI) is a cultural practice in which the amount of irrigation water applied to the orchard is lower than the amount of water loss through evapotranspiration during certain phenological stages when the reproductive development is less sensitive to the occurrence of water stress [8,9,10]. In several fruit crops, such as apricot (*Prunus armeniaca* L.) [11], apple (*Malus domestica* Borkh.) [12], Japanese plum (*Prunus salicina* L.) [13], and sweet cherry (*Prunus avium* L.) [14], among others, RDI has been shown to save up to 50% of irrigation water, reduce excessive vegetative growth, and improve fruit quality at harvest [15]. In European plum trees, the application of late water deficits (LD) has reduced irrigation requirements by up to 40% [6], increased flowering by 17%, and improved dry fruit yield by 14% [16,17]. However, studies on the implementation of RDI in European plums have been conducted exclusively in conventionally managed orchards, as no other cultivation approach has been adopted by the dried plum industry. The lack of innovation in cultivation strategies of European plum trees in Chile contrasts with other fruit crops, such as apple, cherry, almond, and olive, where there has been a higher demand for the development of new planting systems.

The super high-density planting system (SHD) is a relatively recent production strategy for fruit crops that has been employed to increase tree density per hectare and intensify the mechanization of cultural practices [18]. Recently, several plantations of European plum in Chile have been established in SHD (>2000 plants ha^−1^). The effectiveness of this cultivation approach has been demonstrated in a couple of Prunus spp. fruit species, such as almonds (*Prunus dulcis* L.) [19] and Japanese plums (*Prunus salicina* L.) [20]. Yet, there is no scientific information about the productive behavior or the tolerance degree to water stress of SHD European plum orchards. In an SHD olive (*Olea europeae* L.) orchard, a 50% decrease in applied water induced a substantial increase in water stress severity but showed no significant reductions in yield per plant [21].

Trees from SHD orchards are typically grafted on dwarfing rootstocks and trained in continuous hedgerows [22,23] to reduce vegetative growth and control plant size. The increase in the number of trees and the reduced root development of dwarfing rootstocks in SHD systems induce a greater complexity to irrigation management in comparison with conventional low-density orchards. For instance, the use of dwarfing rootstocks can be associated with a shallow root system, reducing the capacity to escape water stress or oxygen deprivation [24,25,26]. On the other hand, several studies have linked the low vigor of fruit trees grafted on dwarfing rootstocks to higher concentrations of abscisic acid (ABA) in shoots [27]. Higher concentrations of ABA may reduce transpiration and improve tolerance to water deficits, as ABA inhibits vegetative growth and induces stomatal closure under water stress [28]. The lack of technical information about how European plum orchards in SHD respond to RDI may limit the adoption of conservative irrigation practices. This is highly relevant for Chilean horticulture, since predictive climate models have projected a substantial reduction in irrigation water availability in those regions where the majority of fruit species are cultivated [29].

Irrigation practices induce transient changes in the soil’s water–oxygen balance that alter the pH of the rhizosphere and stimulate the release of compounds that promote the proliferation of microorganisms that constitute the microbiota [30]. Among these, plant growth-promoting bacteria (PGPB) have attracted great interest in the horticultural industry, as they not only promote plant growth but also improve defense against abiotic stress and acquisition of water and nutrients [31]. Therefore, PGPB are considered relevant inducers of drought resilience in agricultural crops [32]. Under conditions of low water availability, the effects of PGPB’s on plants include changes in root morphology and the synthesis of active osmolytes, antioxidants, phytohormones, extracellular polymers, volatile organic compounds (VOCs), siderophores, and 1-aminocyclopropane-1-carboxylate (ACC) deaminase [33]. However, most studies on the effects of PGPB on drought tolerance in cultivated plants have been conducted in annual species such as cereals and vegetables. In fruit trees, research on plant-bacteria interactions under contrasting irrigation practices has been scarce and inconclusive. For example, the inoculation of walnut plants (*Juglans regia* L.) with Bacillus cereus L90, a PGPB associated with phytohormone synthesis, increased ABA concentration under abundant and deficit irrigation, but did not improve plant tolerance to severe water stress [34].

The Rootpac-20 (*P. besseyi* × *P.cerasifera*) [35] has been the dwarfing rootstock selected for the establishment of new European plum orchards in SHD in Chile. This rootstock has been reported to induce low vegetative growth and high yields per plant [22], low transpiration rates [36], and high fruit dry matter content [37]. However, the degree of drought tolerance of European plum trees grafted on the Rootpac-20 rootstock is unknown. Furthermore, the need to improve the resilience of orchards to abiotic stress and the importance of PGPBs as complementary biological tools for water stress management requires the evaluation of the role that irrigation practices can have on soil microbiota. The aim of this study was to evaluate the effect of RDI on plant water relations, vegetative and reproductive growth, fruit quality, and soil microbial composition in a commercial European plum orchard using the SHD planting system in trees grafted on Rootpac-20.

## 2. Materials and Methods

### 2.1. Description of the Study Site and Weather Data

This study was conducted for two consecutive seasons (2020–2021 and 2021–2022) in a commercial orchard of European plums (*Prunus domestica* L.), French cultivar, located in Peralillo (34°26′27.7″ S 71°25′33.6″ W), Libertador General Bernardo O’Higgins region, Chile. The region’s climate is typified by a Mediterranean climate (Csb), with maximum air temperatures that are generally high during summer (32 °C) and low during winter, ranging from 5 °C to 15 °C. The region’s annual precipitation average ranges from 600 to 800 mm, exhibiting notable seasonal variations in both temperature and precipitation, with the presence of distinct wet and dry seasons. During the spring and summer months, frost events in the study site are relatively rare. However, there were three consecutive days with minimum air temperatures close to 0 °C near the time of fruit set during the 2020–2021 season (October 2nd, 3rd, and 4th). The trees were planted in 2014 and grafted on the Rootpack-20 rootstock (*P. besseyi* × *P. cerasifera*) at a spacing of 3.5 m × 1.5 m in a continuous hedge (canopy dimensions: width of 0.8 m and height of 2.5 m to 3 m). The orchard was drip irrigated with a double line, using two emitters per plant, 0.75 m apart, with an emission rate of 2 L h^−1^ per plant. The orchard was oriented north–south, showing an average fresh yield of 19.5 t ha^−1^ over the last 3 years. The soils belong to the Calleuque series, with a predominantly clayey texture (70% clay, 20% sand, 3% silt) and belong to the Vertisols family (Xererts). The A and B horizons are very clayey soils, with expansive clays and cracks associated with low moisture content. They are very plastic and adhesive soils, with colors ranging from brown to reddish brown, shallow (0.6 m) with low permeability and slow infiltration. The C horizon is dark red, very clayey, and overlies an impermeable layer rich in iron and manganese (hardpan). The plants were pruned twice during the season using a pruning machine (DE800, B.M.V., Sharp Innovators, Alba, CN, Italy). The first pruning was carried out in mid-November and consisted of cutting the hedge with blades, leaving a canopy width of 0.8 m to maintain the plant architecture. The second pruning was carried out at the end of April with saws, leaving a width of 0.6 m to renew the fruiting wood. Harvesting was carried out entirely with a mechanical grape harvester (New Holland VX 7090, CNH Global, Burr Ridge, IL, USA) with a frequency of 380–400 rpm, a working speed of 3 km h^−1^ and a harvesting efficiency of 0.4 ha h^−1^. Pest, weed, and disease management was conducted according to commercial orchard practices. The orchard’s nutritional management followed standard fertigation practices from budbreak to the post-harvest period for European plum trees in Chile. The amounts of nitrogen (N), phosphorus (P_2_O_5_), potassium (K_2_O), calcium (CaO), magnesium (MgO), iron (Fe), manganese (Mn), and zinc (Zn) were determined according to the results of foliar analysis.

### 2.2. Experimental Design

The experimental design was a completely randomized block design with 4 replications. Each experimental unit was a group of 12 plants distributed in 3 adjacent rows (4 plants per row), with physiological measurements taken in the two middle plants of the central row. Two irrigation treatments were applied from the phenological stage of *veraison* (first week of January) (Table 1), with the aim of obtaining differences in plant water status during the fruit ripening period, when water availability is the minimum of the season and the relative growth rate (RGR) of the fruit is low. In the control treatment (WET), commercial irrigation practices were maintained for the whole season, with the aim of satisfying at least 100% of the crop evapotranspiration (ETc) of orchards under conventional management (400–500 trees ha^−1^), trying to maintain an optimum plant water status for European plum, stem water potential (Ψ_stem_) between −1.0 and −0.8 MPa [7]. In the regulated deficit irrigation (LD) treatment, water was completely cut off until Ψ_stem_ values reached a maximum water stress severity of −1.4 MPa (severe water stress) [7]. When the SWP readings of LD plants exceeded −1.4 MPa, irrigation was resumed at the same rate as the control (WET) treatment. When the SWP readings of LD plants were the same as those of WET plants, irrigation was suspended in LD plants. An exception to this treatment management occurred in the 2021–2022 season, during which irrigation was resumed in LD plants for the first time (mid-January) when Ψ_stem_ readings were near −0.9 MPa. This action was taken by the fruit grower to mitigate the potential negative impacts of unusually high temperatures (35 °C) on fruit development.

Before *veraison* and after harvest, commercial irrigation practices were similarly applied in the whole orchard, regardless of the irrigation treatment. Irrigation requirements were calculated based on the estimation of the plum orchard evapotranspiration (ETc = ETo × kc). Crop coefficients (kc) used in this study from budbreak (September) to harvest (mid-February) were obtained from FAO 56 [38] and considered conventional cultivation practices for this fruit crop (400–500 trees ha^−1^ and plant height between 4 and 6 m). In order to estimate ETc values for the SHD orchard, satellite image analysis was employed to facilitate a comparison between the maximum irrigation requirements and the cumulative irrigation applied. The SPIDERwebGIS^®^ (System of Participatory Information, Decision Support and Expert Knowledge for Irrigation River Basin Water Management) platform, developed by the European PLEIADES project [39] and currently operated by AgriSat Iberia (https://www.agrisatwebgis.com/app/spider/webgis/default (accessed on 13 April 2025) and https://www.agrisat.es/en), was used to estimate kc values in the SHD orchard. This platform, in turn, integrates the Plataforma Agrícola Satelital (PLAS) developed by the Instituto de Investigaciones Agropecuarias (INIA) [40,41]. PLAS determines the crop coefficient (kc) as a function of the Normalized Difference Vegetation Index (NDVI) of the crop.

### 2.3. Environmental Conditions

Data on global solar radiation (Wm^−2^), precipitation (mm), reference evapotranspiration (m^3^ ha^−1^), relative humidity (%), and air temperature (°C) were obtained from an agrometeorological station installed in October 2020 at 200 m from the experimental orchard. Sensors were placed between 1.5 and 2 m above the soil surface, and meteorological information was recorded and stored with a sampling frequency of 1 s and storage frequency of 15 min throughout both seasons. Air temperature and relative humidity were recorded with a sensor (HMP60, Vaisala, Helsinki, Finland), wind speed and direction with an anemometer (A100R, Vector Instruments Ltd., Rhyl, North Wales, UK, SF), global radiation with a pyranometer (CM14, Kipp & Zonen, Delft, The Netherlands), precipitation with a rain gauge (ARG100, Campbell Scientific Instrument, Logan, UT, USA). All data were stored in two data loggers (CR10X, Campbell Scientific Instrument, Logan, UT, USA). ETo values were calculated using the meteorological variables recorded with the FAO56 Penman-Monteith daily time step equation (FAO56 P-M) [38].

Volumetric soil water content was measured across the effective rooting zone of the plants only in the first block of the orchard during both seasons from one month before the onset of fruit maturity at veraison (mid-January) to the beginning of leaf senescence (end of March) (Table 1) during the first and second seasons. Evaluations were made with capacitance sensors (GS1, Decagon devices, Pullman, WA, USA) installed in the center row at 0.75 m from the plant, at two depths (−0.3 and −0.6 m). The effective rooting zone and the sampling depth were determined based on the root system development in four soil pits randomly distributed throughout the SHD orchard. Data were recorded and stored every 15 min in two dataloggers (Em5b and Em50, Decagon devices, Pullman, WA, USA). Four volumetric water meters (Dishnon, Arad Ltd., Dalia, Israel), one per block, were installed at the beginning of each irrigation line to estimate the amount of water irrigated on 1 October 2020.

Photosynthetically active photon flux density (PPFD, µmol m^−2^ s^−1^) and leaf area index (LAI) were determined weekly using a ceptometer (LP-80, Decagon Instruments, Washington, DC, USA) with four measurements for each plant sampled at midday. Measurements were taken at 0.2 m below the plant canopy to estimate the internal PPFD, with each measurement taken at 0.05, 0.20, 0.30, and 0.40 m from the trunk. The outer PPFD was estimated in the inter-row at 1.75 m from the plant and at 1.6 m above the ground.

### 2.4. Plant Water Status, Physiology, and Growth

Severity of plant water stress was determined weekly from budbreak to harvest in both seasons by measuring midday stem water potential (Ψ_stem_) in two leaves per sampled plant, selected from the shaded part of the canopy and without visual symptoms of biotic or abiotic stress. Measurements were performed between 12:00 and 15:00 h using a pressure chamber (PMS-615, PMS Instruments, Portland, OR, USA). Sampled leaves were previously covered with an opaque airtight bag for at least 40 min according to the method described by McCutchan and Shackel [7]. Stomatal conductance (mmol m^−2^ s^−1^) was determined simultaneously with stem water potential measurements using a steady-state porometer (SC-1, Decagon devices, Washington, DC, USA) on three mature sun-exposed leaves per sampled tree from the apical third of the shoots. Photosystem II (PSII) efficiency was determined as Fv/Fm using a chlorophyll fluorescence meter (Pocket PEA, Hansatech Instruments, Norfolk, UK). Both measurements were made at midday, once a week, during both seasons. To determine the photochemical efficiency of PSII, three randomly selected apical sun-exposed leaves per plant were dark-adapted for 30 min using leaf clips [42] prior to measuring the minimum fluorescence (*Fo*) and maximum fluorescence (*Fm*). The variable fluorescence (*Fv*) was then determined as the difference between *Fm* and *Fo*. Photosystem II efficiency (*Fv*/*Fm*) was calculated using the following relationship:

(1) $$\frac{\mathrm{Fv}}{\mathrm{Fm}}=\frac{\mathrm{Fm}-\mathrm{Fo}}{\mathrm{Fm}}$$

### 2.5. Yield Components and Fruit Quality

Yield and fruit quality data were recorded in the two central plants for each treatment block combination. The percentage of fruit drop in relation to the total yield at harvest was estimated by manually counting the fruit on the ground before harvest. This was performed by considering a quadrant of the size of the planting frame (5.25 m^2^) in the two plants evaluated in each block treatment combination. At the time of harvest maturity for dehydrated fruit (24–25 Brix), the fruit of each sampled tree was manually harvested. At the time of harvest, the crop load of each sampled tree (kg plant^−1^) was estimated, and the total number of fruits was counted. The total weight per tree was then determined using a platform scale. Subsequently, a random sample of 100 fruits was selected from each plant, stratified by block treatment. From this sample, a random subsample of 50 fruits was selected for analysis of fresh fruit quality, and an additional 50 fruits were selected for analysis of dried fruit quality. In the initial subsample for fresh fruit quality, the individual fresh weight of each fruit (g) was determined using a precision balance with an accuracy of ±0.1 g (APTP457A, Electronic Scale balance, Kuala Lumpur, Malaysia). Flesh firmness (lbf) was determined using a digital penetrometer with an 8 mm plunger (FM200, PCE Instruments, Southampton, UK). This measurement was taken at the midpoint of the longest side of the fruit after manual removal of a section of skin. The color of the pulp was then determined in a slice taken from each fruit using a portable colorimeter (CR-10, Konica Minolta, Tokyo, Japan). The color was determined within the CIELAB color space, expressed as the coordinates L*a*b*. L* represents lightness, a* represents red/green, and b* represents yellow/blue. The concentration of soluble solids (Brix) in the juice, extracted from each fruit by manual means, was determined using a digital refractometer (HI 96801, Hanna Instruments, Woonsocket, RI, USA). Prior to each measurement, the refractometer was calibrated with distilled water. The dry subsample was transferred to dehydration ovens, where the fruit was placed on trays and exposed to 85 °C for 19 h, resulting in a reduction in the fruit’s moisture content from 85% to 19%. Subsequently, the dried fruits were individually weighed, and the drying rate per treatment was calculated using the ratio of the fresh weight to the dry weight. Flower return was conducted at full bloom (early September) (Table 1), and flowers were counted on four branches per tree in the two trees monitored per treatment block combination. Flower return was expressed as the number of flowers per meter squared.

### 2.6. Root System Characterization

During the period of maximal floral development (September), the number and size of roots in one block were determined for both irrigation treatments. A soil pit measuring 1 m in depth and width was excavated in the center of the center row. A grid of the same dimensions as the soil pit (1 m^2^) was divided into 100 grids of 0.1 m^2^. Prior to counting, the soil pit was meticulously cleaned with an agrological knife in order to expose the root system. Each visible root was classified according to diameter, with roots measuring less than 0.5 mm classified as fine, those between 0.5 and 2.0 mm classified as thin, those between 2.0 and 5.0 mm classified as medium, those between 5.0 and 7.0 mm classified as medium to coarse, and those greater than 7.0 mm classified as coarse. The proportion of roots of varying diameters to the total number of roots sampled in each test soil pit was determined.

### 2.7. Evaluations of the Cultivable Soil Microbiota

During the first week of September of the last season, the populations of four groups of bacteria (*Azotobacter* spp., *Azospirillum* spp., Actinobacteria, and anaerobic bacteria) were evaluated to characterize the effect of irrigation on the cultivable soil microbiota. The sampling time was chosen to evaluate whether irrigation practices applied at the end of the previous season were able to alter the soil microbiota that will interact with plant growth at the beginning of the following season. For this purpose, soil samples were collected with an auger from the effective rooting zone of the plants in three of the four blocks evaluated, at the same locations where the soil moisture probes were installed, taking two soil samples per treatment-block combination at 0.2 and 0.4 m depth. Composite samples of 250 g of soil, including free and rhizospheric soil, were collected. Prior to each sampling, the materials used were disinfected in 70% ethanol. Each composite sample was placed in a polyethylene bag, previously identified according to the number of samples, cultivar, and density. Finally, the bags containing the samples were sealed and placed in a refrigerated box (5 °C) until they were analyzed in the laboratory. Each soil sample was homogenized to select a 10 g subsample, which was dissolved in 100 mL of sterile saline solution (0.89% NaCl) in an Enlenmeyer flask. The flasks containing the dilution were kept on an orbital shaker at 150 rpm with constant agitation for two hours. Dilutions of 10 were made from the suspension until the 10^−5^ dilution was reached. Dilutions ranging from 10^−2^ to 10^−5^ were used for inoculation into the different culture media, depending on the microbial group to be quantified. In addition, for the determination of the dry weight of the soil, a subsample of 50 g of each experimental unit was placed on metal plates and placed in a dry air oven at 60 °C until the weight was constant. All culture media were prepared in deionized water and autoclaved at 120 °C/1 atm for 20 min. After sterilization, they were distributed in Petri dishes with 10 mL of medium in each one. This procedure was performed under aseptic conditions in a laminar flow chamber. The methodology used to count all the populations is based on the distribution of an aliquot of 100 µL of each dilution on the surface of the agar using a sterile glass rod. The plates were incubated at 25 ± 2 °C in the dark for 3 days, except for the Congo Red medium, which was incubated for 7 days before counting the colony-forming units (CFU). For counting, dilutions were chosen in which the number of colonies was between 30 and 300. For strict anaerobic bacteria, standard nutrient agar medium (MERCK) was used and incubated under anaerobic conditions in the GasPak™ EZ Anaerobe Container System Sachets chamber. Actinobacteria were enumerated on Jensen agar (2 g L^−1^ dextrose, 0.2 g L^−1^ casein; 0.5 g L^−1^ K_2_HPO_4_, 0.2 g L^−1^ MgSO_4_·7H_2_O, trace FeCl_3_·6H_2_O, 2.5% (*w v*^−1^) agar). Bacteria of the genus *Azotobacter* were isolated on LG medium [43] and those of the genus *Azospirillum* on RC selective medium [44]. The relative amount of bacteria was determined as the ratio between the CFU of each group and the sum of the CFU of the four groups evaluated.

### 2.8. Statistical Analysis

The data were subjected to an analysis of variance (ANOVA) after testing for normality distribution (Shapiro–Wilk), homogeneity of error variances (Levene’s test), and additivity (Tukey). Differences between means were determined using the LSD test (alpha = 0.05). The relationship between physiological variables and root development measures was analyzed using linear and quadratic regression analysis. All statistical analyses were performed using the statistical software SAS 9.4 (SAS Studio, University Edition, SAS Institute, Cary, NC, USA).

## 3. Results

### 3.1. Environmental Conditions and Characterization of Irrigation

The amount of irrigation for the WET plants was about 8500 m^3^ ha^−1^ in the first season, while the irrigation of the same treatment increased by about 40% in the second season (Table 2). The estimated cumulative evapotranspiration of the SHD orchard for the first and second seasons was 5204 and 5288 m^3^ ha^−1^, respectively. The amount of water applied in the first and second seasons was 9219 and 12,423 m^3^ ha^−1^, respectively, with water savings of about 23% in plants under LD in both seasons. In general, plants under LD had a clear effect on water productivity, increasing values between 40% and 43%.

**Figure 1:**
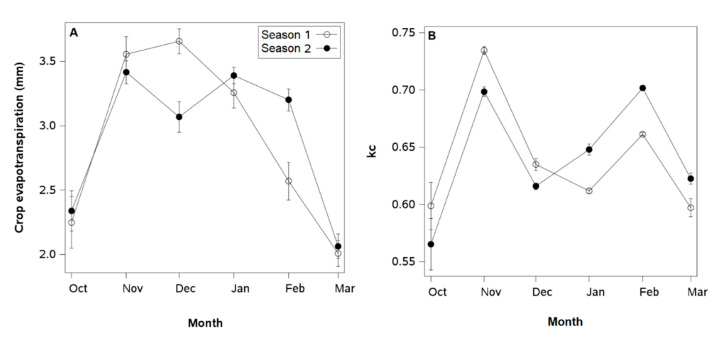
Monthly average of (**A**) crop evapotranspiration and (**B**) crop coefficient in a European plum orchard in super high density (SHD) in Peralillo, O’Higgins Region, from anthesis to the onset of leaf senescence (October to March) during the 2020–2021 and 2021–2022 seasons. Error bars represent ±1 se.

Seasonal differences in monthly ETc (close to 0.5 mm) were observed in December and February (Figure 1A). In the first season, the maximum evaporation rate of 3.5 mm was recorded in December, followed by an abrupt 40% decrease in monthly ETc in March. The maximum crop coefficients (kc) were reached in November in both seasons and were very close to 0.7 (Figure 1B). In December, the month in which mechanized training pruning was carried out, kc was reduced by 15%. In February, kc returned to values relatively similar to those before pruning. However, the kc of the first season in February was slightly lower (5%) than that of the second season.

**Figure 2:**
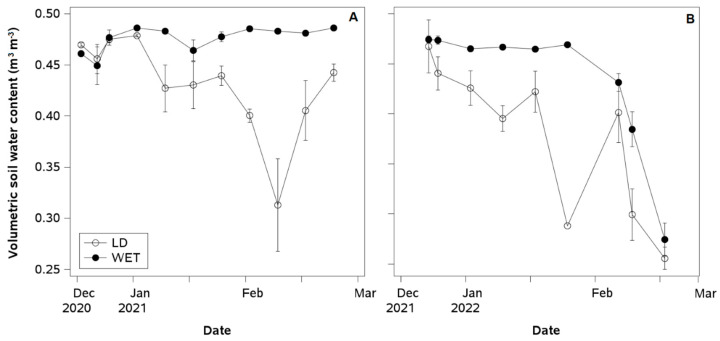
Weekly values of volumetric soil water content over two depths (−30 and −60 cm) from fruit ripening to the onset of leaf senescence (December to March) in a European plum orchard cv. French in super high density (SHD) under two irrigation treatments (LD: late water deficit and WET: commercial irrigation) in Peralillo, O’Higgins Region, during the (**A**) 2020–2021 and (**B**) 2021–2022 seasons.

In WET and LD plants, soil volumetric water content (SWC) was close to 0.5 m^3^ m^−3^ before the first irrigation cutoff in January (Figure 2A,B). For the first irrigation cutoff, early January 2020 and late December 2021, SWC values decreased to 0.4 m^3^ m^−3^ in LD plants. For the second cutoff in late February, SWC reached 0.3 m^3^ m^−3^ in LD plants. A third irrigation cutoff in the second season reduced SWC in LD plants from 0.4 to 0.3 m^3^ m^−3^ in just one week. At the end of the second season, both irrigation treatments showed a decrease in SWC, reaching values close to 0.25 m^3^ m^−3^.

### 3.2. Physiological Responses

**Figure 3:**
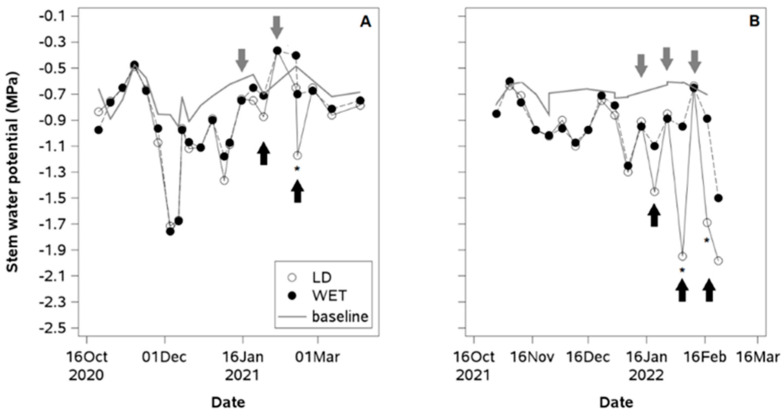
Stem water potential at midday (12:00–15:00 h) in a European plum orchard in super high density (SHD) under two irrigation treatments (LD: late deficit and WET: commercial irrigation) in Peralillo, O’Higgins Region, during the (**A**) 2020–2021 and (**B**) 2021–2022 seasons. Gray arrows indicate the date of irrigation cutoff in LD plants, black arrows indicate the date of irrigation resumption in LD plants. The gray line without markers indicates the baseline optimum water status for European plum [7]. Asterisks indicate significant differences (*p* ≤ 0.05, n = 4).

**Figure 4:**
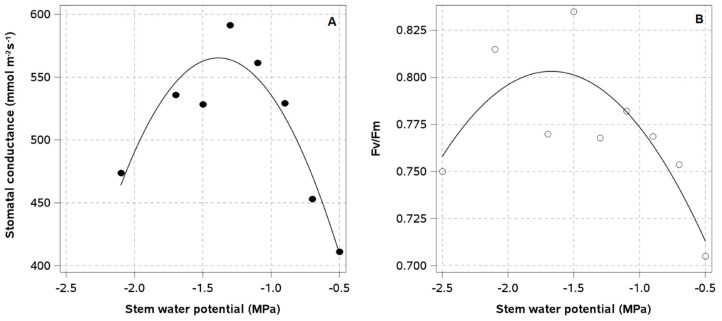
Relationship between midday stem water potential (12:00–15:00 h) and (**A**) leaf stomatal conductance (R^2^: 0.74; *p*-value < 0.05; Y = −198.6x^2^ − 550.6x + 183.7) and (**B**) *Fv*/*Fm* (*p*-value < 0.05; R^2^ = 0.66; y = −0.66x^2^ − 0.219x + 0.62) in a European plum orchard in super high density (SHD) under two irrigation treatments (LD: late water deficit and WET: commercial irrigation) in Peralillo, O’Higgins Region, during the 2020–2021 and 2021–2022 seasons.

Measured Ψ_stem_ values for both irrigation treatments were between 2.0 and 0.5 MPa near the SWP baseline until the first irrigation cutoff (Figure 3A,B). After treatment application, LD plants exhibited reductions in Ψ_stem_ in both seasons, but measured Ψ_stem_ values for both irrigation treatments were consistently lower in the second season. In the first season, LD plants reduced their Ψ_stem_ to −0.8 MPa two weeks after the first irrigation cutoff, but there were no significant differences between irrigation treatments. During the second irrigation cutoff, plants reached a Ψ_stem_ of −1.2 MPa after 12 days, with a difference of −0.5 MPa from the WET (Figure 3A). In the second season, LD plants showed a Ψ_stem_ of −1.5 MPa one week after the first irrigation cutoff. After the second irrigation cutoff, the maximum water stress severity reached −2.0 MPa, while for the third cutoff, the Ψ_stem_ reached −1.7 MPa (Figure 3B). The regression analyses showed that Ψ_stem_ values near −1.5 MPa were associated with the maximum seasonal values of g_s_ (Figure 4A) and *Fv*/*Fm* (Figure 4B). Once plants reached a −2.0 MPa Ψ_stem_, g_s_ decreased by 20% (Figure 4A), while *Fv*/*Fm* values remained close to the maximum of 0.8 (Figure 4B). Plants with Ψ_stem_ values above −0.5 MPa experienced a 30% decrease in g_s_ and a 20% decrease in *Fv*/*Fm*.

### 3.3. Yield and Fruit Quality Estimates

**Figure 5:**
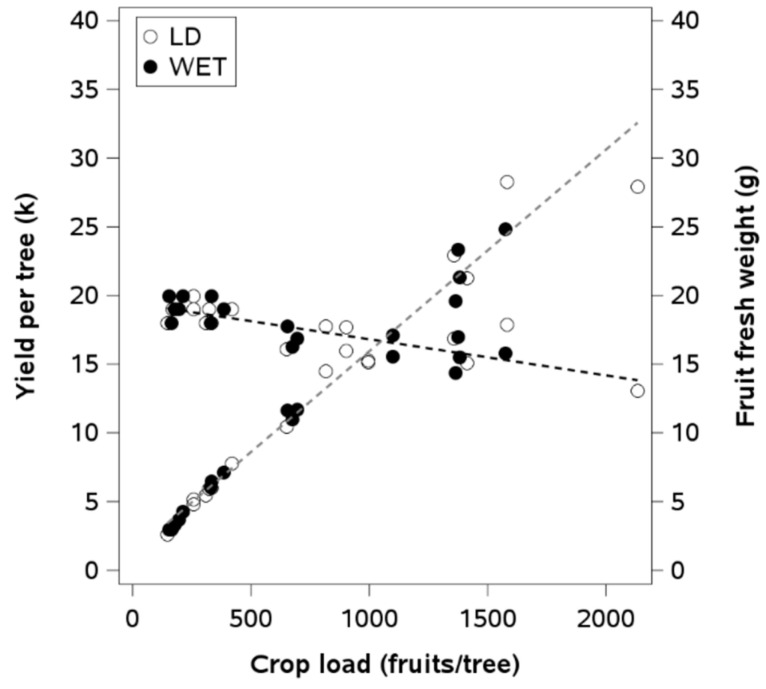
Linear regressions between fruit load with yield per plant (y = 1.27 + 0.015x (*p*-value < 0.0001; R^2^ = 0.97; n = 32) and with fresh weight of an individual fruit (y = 19.4 − 0.0026x (*p*-value < 0.0001; R^2^ = 0.67; n = 32) in a European plum orchard in super high density (SHD) under two irrigation treatments (LD: late water deficit and WET: commercial irrigation) in Peralillo, O’Higgins Region, during the 2020–2021 and 2021–2022 seasons.

The yield of fresh fruit per plant was not affected by LD. The average yield for the first and second seasons was 9.3 and 32.3 tons per hectare, respectively (Table 3). The number of fruits per tree was not significantly affected by irrigation treatment in either season. The mean number of fruits per tree was 260 and 1065 in the first and second seasons, respectively. There were no treatment differences in the number of flowers (Table 3). The fresh fruit quality parameters were similar between the irrigation treatments in both seasons (Table 4). The soluble solids concentration and the fresh weight were 5 Brix and 2 g higher in the first season. There was a linear relationship between crop load in fresh weight per fruit and yield per plant (Figure 5).

### 3.4. Root System Response and Cultivable Soil Microbiota

**Figure 6:**
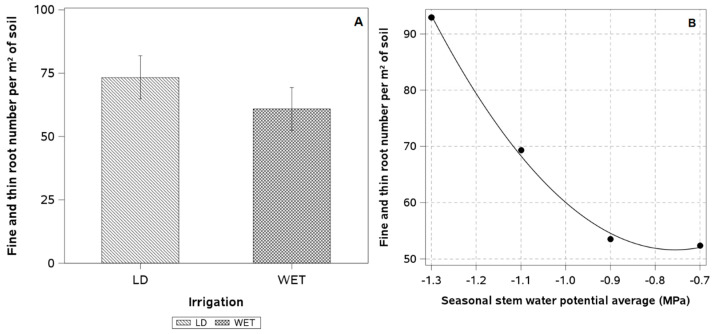
(**A**) Number of fine and thin roots per soil area and (**B**) quadratic regression between the seasonal average of stem water potential and the number of fine and thin roots per square meter of soil in two blocks of a European plum orchard in super high density (SHD) under two irrigation treatments (LD: late deficit irrigation and WET: commercial irrigation) in Peralillo, O’Higgins Region during the 2021–2022 season). Error bars represent ±1 se.

The plants showed a poorly developed root system at a maximum depth of 60 cm. Of the total roots, 90% were fine and thin. LD plants had almost 20% more fine and thin roots than WET plants (Figure 6A). Regression analysis revealed a clear quadratic relationship between the average seasonal stem of plants and the number of fine and thin roots per m^2^ of soil (Figure 6B).

**Figure 7:**
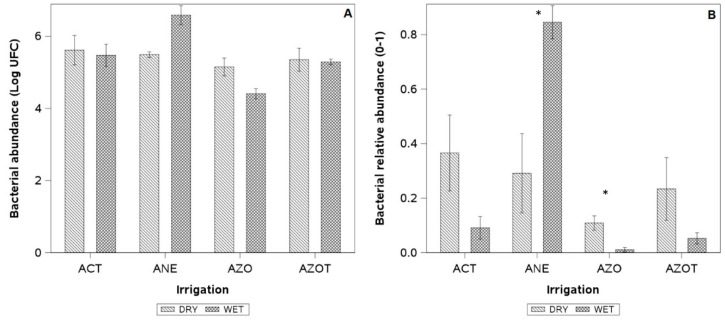
(**A**) Absolute content (logarithm of colony forming units, CFU) and (**B**) relative abundance of bacteria in the soil in a European plum orchard in super high density (SHD) under two irrigation treatments (LD: late deficit irrigation and WET: commercial irrigation) in Peralillo, O’Higgins Region during the 2020–2021 season. ACT = Actinobacteria; ANE = strict anaerobic bacteria; AZO = bacteria of the genus *Azospirillum*; AZOT = bacteria of the genus *Azotobacter*. Asterisk indicates significant differences (*p* ≤ 0.05, n = 3). Error bars represent ±1 se.

The application of LD had no statistical effect on the total UFC, but it did affect the relative abundance of some groups of cultivable microorganisms in free soil (Figure 7A,B). The relative abundance of *Azospirillum* spp. bacteria in soils under LD were ten times higher than those under the WET (Figure 7B). While there were no significant differences overall, the relative contents of the genus Azotobacter and the phylum Actinobacteria were three and eight times higher, respectively, under LD. Conversely, soils under the WET exhibited a relative abundance of anaerobic bacteria close to 90%, which was three times higher than that observed in soils under the LD treatment. It should provide a concise and precise description of the experimental results, their interpretation, as well as the experimental conclusions that can be drawn.

## 4. Discussion

The results of this study prove that European plum orchards established in SHD and grafted on the dwarfing rootstock Rootpac-20 can be deficit irrigated without reducing fresh and dry yields. Applying a late water deficit resulted in significant water savings, with an improvement in water productivity of approximately 40% in both seasons. The period of the late water deficit application coincides with the time of the year when (1) the atmospheric evaporative demand is at its maximum and (2) the availability of irrigation water is at its lowest in Central Chile. The water savings generated by LD during this period are of great significance, as they increase the water availability to irrigate fruit species that are more sensitive to water stress than the European plum. These results contradict those reported by McCutchan and Shackel [7]. Their findings indicated that severe late water deficits (Ψ_stem_ < −1.5 MPa) had no effect on dry yield but resulted in a 4 t ha^−1^ reduction in fresh yield. The lack of irrigation effects on fresh and dry yield was the result of a combination of factors, including the following: (A) The application of LD at the onset of *veraison* in January did not coincide with any phenological event that determined the number and weight of fruits. For example, the number of fruits per tree is largely determined by the number of flowers in the current season, which is defined during bud induction and initiation in late November of the previous season [45]. Furthermore, drupes reach their maximum relative growth rate (RGR) during the first few weeks after fruit set [46]. Any significant alteration of physiological processes, such as stomatal conductance or PSII photochemical efficiency, that may alter carbohydrate supply rate during fruit set will inevitably result in a discrepancy between the actual and potential RGR, which in turn will lead to a reduction in final fruit size and yield at harvest. Since the severe water stress in LD plants occurred between one and two months away from bud induction and fruit set, both phenological stages occurred under optimal water conditions, regardless of the irrigation treatment. (B) Even though previous studies in vigorous orchards have shown a reduction near 60% in the photosynthesis rate of severely water-stressed European plum trees [47], the transient occurrence of moderate (Ψ_stem_~−1.2 MPa) to severe water stress (Ψ_stem_~−2.0 MPa) in low-vigor trees grafted on the Rootpac-20 rootstock had no significant impact on parameters that alter net assimilation rates, such as g_s_ and *Fv*/*Fm*. When plants reached a Ψ_stem_ of −2.0 MPa, gs was reduced by 20%, but *Fv*/*Fm* remained close to the maximum values recorded for European plum trees (*Fv*/*Fm*~0.8) [48]. These findings are inconsistent with those reported by Lampinen et al. [47], which indicated about a 50% reduction in g_s_ and photosynthesis in plants of cv. French when Ψ_stem_ reached −1.7 MPa. In almond trees (*Prunus dulcis* L.) grafted on Rootpac-20, only the application of a very severe level of water stress (Ψ_leaf_ ≤ −2.0 MPa) reduced g_s_ to values that compromised the photosynthesis rates of leaves [49]. Since dwarfing rootstocks have been reported to exhibit higher concentrations of ABA in shoots [27], it is possible that the grafting of *Prunus* spp. plants onto a dwarfing rootstock, such as Rootpac-20, may induce hormonal changes associated with modifications in the stomatal response to water stress in comparison to that reported in previous studies with plants grafted on rootstocks of higher vigor. (C) The crop loads of the plants in SHD were at least four times lower than those reported in previous irrigation studies for conventional European plum orchards. In the present study, the application of LD did not affect yield estimates or any fruit quality parameter, suggesting that moderate, severe, and very severe water stress showed little impact on the carbohydrate supply to fruits during stage III of fruit growth and development. This finding highlights the importance of the competition for carbohydrates among fruits as a key determinant of the impact of water stress on yield and fruit quality. The fruit load in the orchard in SHD did not exceed 1200 fruits, whereas in a conventional production system with lower planting densities, fruit loads are usually higher than 5000 fruits. From a commercial perspective, the lack of differences in fruit quality and yield between irrigation treatments is of greater consequence nowadays, given the increasing attractiveness of exporting fresh fruits from European plum trees to the Asian market in Chile.

Despite the lack of irrigation effects on fruit size parameters, the equatorial diameter and fruit weight did not reach the commercial optimum values required by the fresh and dry markets (33 mm and 20–23 g, respectively). Although the crop load of the first season was reduced to 259 fruits per tree^−1^ by a spring frost close to flowering, both irrigation treatments exhibited the lowest fruit sizes in the first season. Previous studies found that the relationship between crop load and fruit size was quadratic in European plum trees, with the highest slope value for this relationship observed at low crop loads [6]. In this study, the relationship between crop load and fresh fruit weight was linear for the range between 0 and 1200 fruits per plant, suggesting that competition for assimilates among fruits was not a limiting factor for fruit growth in plants with a low crop load. A failure in the irrigation system that lasted two weeks and induced severe levels of water stress (Ψ_stem_ ~−1.7 MPa) near the fruit set may have been the main factor that explains the low fruit size in the first season. In the second season, the 312% increase in crop load was probably the primary cause of the small fruit size at harvest. A substantial increase in crop load in the second season was expected for European plum trees with unusually low crop loads in the preceding season, as this species is considered a plant with a pronounced alternate bearing behavior [17].

European plum trees grafted on Rootpac-20 would have a higher photosynthetic risk in over-watered conditions than under moderate or severe water stress. Plants that exhibited Ψ_stem_~−0.5 MPa were near or above the SWP baseline for well-irrigated conditions, inducing decreases of 30% in g_s_ and 20% in *Fv*/*Fm*. These reductions may be related to poor oxygenation of roots subjected to abundant irrigation [50,51]. The regulation of stomatal opening and closing under conditions of excess moisture has been attributed to both an increase in abscisic acid (ABA) concentration and a decrease in cytokinins and gibberellins in roots [52,53]. Reductions in physiological importance in g_s_ and *Fv*/*Fm* were observed in apple plants grafted on the M9 dwarfing rootstock after 30 days under hypoxic conditions [54]. In the present study, the amount of irrigation water applied throughout the season to plants in the SHD of the WET was calculated to satisfy the irrigation needs of conventionally managed European plum orchards (between 9000 and 12,000 m^3^ ha^−1^) [16]. However, the analysis of satellite images and NDVI estimates for the SHD orchard showed that the total evaporative demand for the first and second seasons was 5204 and 5288 m^3^ ha^−1^, respectively. Therefore, the quantity of irrigation water applied in the SHD orchard would have exceeded the amount necessary to meet at least twice the water demand in both seasons, which clearly showed that plants were consistently under overirrigation conditions. The objective of irrigation in conventionally managed European plum orchards is to meet the evaporative demand of tall trees (between 5 and 6 m in height) with canopies that cover the ground almost entirely. This results in maximum crop coefficients close to 1.0 [55]. In this study, plant canopies in SHD did not completely cover the 3.5 m interrow, as the canopy width was between 0.8 and 0.9 m using trimming machines. As a result, approximately 75% of the orchard area was without vegetation during the growing season.

The impact of overirrigation in the SHD orchard was more evident in the WET, as its average soil volumetric water content was closer to saturation (0.5 m^3^ m^−3^) than field capacity (FC) (0.45 m^3^ m^−3^) during the experimental period in both years. Despite an increase of 40% in irrigation water applied, water stress severity was higher in WET and LD plants during the second season. In the case of WET plants, Ψ_stem_ values indicated optimal plant water conditions during the first season. Conversely, in the subsequent season, Ψ_stem_ values of WET plants represented mild to moderate water stress levels for European plum, ranging from −1.2 to −1.0 MPa [6]. Plants under LD in the first season reached moderate water stress levels for European plum for approximately one week, with Ψ_stem_ values ranging from −1.4 to −1.2 MPa [6]. In contrast, water stress severity in the second season was moderate for the first irrigation cutoff but very severe for the second cutoff (<−1.7 MPa) [6]. Consequently, applying 40% more irrigation water in the second season caused no increase in water stored in the effective rooting zone, highlighting how unnecessary overwatering is as an irrigation practice to avoid water stress. The higher severity of water stress registered by both treatments during the second season mostly reflected the influence of higher evaporative water demand, as the monthly average ETc during February of the second season, when the most severe Ψ_stem_ drops occurred, was approximately 30% higher than that observed during the same month of the previous year. The observed increase in ETc during February of the second season was likely due to two primary factors: (1) higher VPD and (2) slightly higher kc values resulting from the stimulation of sprouting following the spring pruning in November. Yet, previous research has indicated that the decline in Ψ_stem_ in LD treatments may be less pronounced and less severe when irrigation is partially restricted rather than fully withheld [6].

The application of LD with a complete irrigation cutoff, as opposed to a proportional decrease in irrigation amount or frequency, reduced SWC to values close to PMP (0.25 m^3^ m^−3^). However, once plants reached moderate to severe levels of water stress, LD plants were irrigated as WET plants. The transient and abrupt processes of severe drying and rehydration of soils caused changes in microbiological dynamics, as previously stated by Meisner et al. [56]. Soils subjected to LD tended to show higher relative abundances of bacterial genera and phyla adapted to aerobic conditions and regarded as beneficial microorganisms for plants [57,58]. For instance, the relative abundance of *Azospirillum* spp. was approximately tenfold higher in the LD treatment. Despite the lack of significant differences between irrigation treatments, the relative abundance of the genus Azotobacter and the phylum Actinobacteria was three and eight times higher in the LD treatment. *Azospirillum* is a bacterial genus that, like *Azotobacter*, is characterized by nitrogen fixation and the production of phytohormones [58] that are often associated with nitrogen and water scarcity [59]. On the other hand, Actinobacteria, known for their role as plant symbionts and biological control agents, have the potential to increase their population under conditions of low soil moisture content [60,61]. The reduction in soil moisture in plots under the LD treatment can kill a substantial number of bacteria, releasing high quantities of organic matter within the effective rooting zone of plants [62]. After irrigation restarted in LD plants, soil water content increased to values near field capacity, solving organic matter and increasing bacterial populations to a greater extent than in the soils maintained at high moisture levels in the WET [63]. Soils subjected to the WET exhibited a relative abundance of anaerobic bacteria near 90%, approximately threefold higher than that observed in soils under the LD treatment. Soil conditions in the WET may have stimulated the proliferation of anaerobic bacteria, outcompeting the populations of obligate aerobic bacteria. The shallow root system and the low leaf area limited the water uptake capacity of dwarfing rootstocks, which probably modulated the magnitude of the irrigation effects on *Azospirillum* and anaerobic bacteria population changes.

The higher relative abundance of some groups of beneficial soil bacteria in the LD treatment may explain the higher number of fine and thin roots in plants subjected to transient periods under water stress. These kinds of bacteria stimulate root growth and phytohormone synthesis, such as indole acetic acid and gibberellins [58,64]. The stimulation of root growth improves the adaption to drought by increasing the quantity of fine and thin roots, which are the ones that exhibit the highest rates of water and nutrient uptake and represent nearly 90% of all roots sampled. Furthermore, the highly significant correlation between season-average stem water potential and the total number of fine and thin roots confirms that transient moderate water stress stimulates root development, while very high humidity conditions may reduce it. The findings of the present study suggest that European plum trees grafted on a dwarfing rootstock in an SHD orchard can be effectively deficit-irrigated to enhance tolerance to a wide range of water stress severities, not only through stomatal regulation and root development stimulation, but also through the maintenance and preferential reproduction of specific beneficial soil bacteria. Surprisingly, the microbiological response to regulated deficit irrigation was statistically significant even nine months after its application, following the winter rainy season (>400 mm year^−1^) and three weeks before the beginning of the following season in spring.

## 5. Conclusions

The findings of the present study demonstrated that regulated deficit irrigation can be successfully applied in European plum orchards under the super high-density (SHD) production system, exhibiting no decline in yield per plant or fresh and dry fruit quality. Deficit-irrigated plants exhibited moderate to severe water stress, but their leaves maintained high levels of stomatal conductance and photochemical efficiency. The successful application of regulated deficit irrigation in European plum trees in the SHD system represents a substantial advancement for the Chilean European plum industry, as fruit growers can save high amounts of irrigation water (up to 40%) in areas affected by severe and prolonged droughts. Although the LD treatment induced transient reductions in soil moisture to values near the permanent wilting point, there was an increase in the relative population of beneficial plant growth-promoting bacteria and the number of fine roots. Therefore, the SHD system, combined with RDI, not only maximizes water use efficiency but also promotes soil resilience to drought and sustainable orchard water management. This study provides a strong foundation for future investigations and the potential adoption of RDI practices in modern high-density orchard systems.

## Figures and Tables

**Table 1:** Date of occurrences of several phenological stages of a SHD European plum orchard in Peralillo, O’Higgins Region, Chile during the 2020–2021 and 2021–2022 seasons.

Phenological Stages *	Seasons	
	2020	2021
Bloom	25 September	30 September
Budbreak	29 September	4 October
Fruit set	5 October	7 October
Pit hardening	7 November	11 November
Veraison	17 January	20 January
Harvest	18 February	23 February

* The date of occurrence of each phenological stage was recorded when the typical visual characteristics of that stage were observed in at least 50% of the evaluated plants.

**Table 2:** Cumulative values of irrigation, precipitation, applied water (irrigation + precipitation) and water productivity in a European plum orchard in super high density (SHD) under two irrigation treatments (LD: late water deficit and WET: commercial irrigation) in Peralillo, O’Higgins Region, during the 2020–2021 and 2021–2022 seasons.

Cumulative Values	Season 2020–2021			Season 2021–2022		
	WET	LD	Diff	WET	LD	Diff
Crop evapotranspiration (m^3^ ha^−1^)	5204			5288		
Irrigation (m^3^ ha^−1^)	8544	6591	23%	11,991	9097	24%
Precipitation (m^3^ ha^−1^)	672			432		
Applied water (m^3^ ha^−1^)	9216	7263	23%	12,423	9529	23%
Water productivity (kg m^−3^)	1.1	1.6	45%	3.0	4.2	40%

**Table 3:** Orchard and tree yield, number of fruits per tree, floral return, fallen and split fruit at harvest in a super high density (SHD) European plum orchard subjected to two irrigation treatments (LD: late deficit irrigation and WET: commercial irrigation) during the 2020–2021 and 2021–2022 seasons. NS means not significantly different, n = 8.

Production	Irrigation Treatment	
	WET	LD
***2020–2021***		
Orchard yield (ton ha^−1^)	8.8	9.8
Plant yield (kg treel^−1^)	4.6	5.1
Crop load (fruits tree^−1^)	243	275
Return bloom (flowers m^−1^)	101	115
Falling fruits (%)	25.3	33.9
***2021–2022***		
Orchard yield (ton ha^−1^)	30.4	34.1
Plant yield (kg treel^−1^)	15.9	17.9
Crop load (fruits tree^−1^)	1001	1130
Return bloom (flowers m^−1^)	24	22
Falling fruits (%)	10.8	11.3

**Table 4:** Fruit quality parameters at harvest in a super high-density (SHD) orchard subjected to two irrigation treatments (LD: late deficit irrigation and WET: commercial irrigation) during the 2020–2021 and 2021–2022 seasons.

Harvest Quality	Irrigation Treatment	
	WET	LD
***2020–2021***		
Split fruit (%)	4.3	4.8
Fresh weight per fruit (g)	18.8	18.2
Equatorial diameter (mm)	26.8	26.6
Polar diameter (mm)	33.0	32.0
Pulp firmness (lbf)	2.0	2.4
Soluble solids concentration (Brix)	25.0	25.1
*Color*		
L	31.9	34.6
a	11	11.8
b	28.7	30.7
Conversion ratio	2.8	3.4
***2021–2022***		
Split fruit (%)	4.0	4.3
Fresh weight per fruit (g)	16.1	16.2
Equatorial diameter (mm)	28.0	27.5
Polar diameter (mm)	34.8	34.5
Pulp firmness (lbf)	1.4	1.4
Soluble solids concentration (Brix)	20.1	21.3
*Color*		
L	29.2	29.0
a	10.5	10.2
b	28.2	28.4
Conversion ratio	3.6	3.7

## Data Availability

The original contributions presented in this study are included in the article. Further inquiries can be directed to the corresponding author.

## References

[1] Chile Prunes Production Analysis of the 2022–2023 Season. X Expo Dried Plums: The Reunion of the Industry. Santiago, Chile. https://chileprunes.cl/2023/03/23/at-the-reunion-of-the-prunes-industry-a-key-objective-is-set-to-return-to-india/?lang=en.

[2] ODEPA Fruit Cadaster: Main Results. Region of Ñuble. September 2022. https://bibliotecadigital.odepa.gob.cl/handle/20.500.12650/71983.

[3] Moreno C., Granger C., Flores C., Jara R., Manríquez R. (2020). Ciruelas D’Agen para la exportación en fresco, el desafío de cosechar en la madurez adecuada. Rev. Frutícola.

[4] Korkmaz K., Bolat I., Uzun A., Sahin M., Kaya O. (2023). Selection and Molecular Characterization of Promising Plum Rootstocks (*Prunus cerasifera* L.) among Seeling-Origin Trees. Life.

[5] Meland M. (2005). High density planting systems of European plums the effect of growth and productivity of three cultivars after nine years. Acta Agric. Scand. Sect. B Soil Plant Sci..

[6] Lampinen B.D., Shackel K.A., Southwick S.M., Olson W.H. (2001). Deficit irrigation strategies using midday stem water potential in prune. Irrig. Sci..

[7] McCutchan H., Shackel K.A. (1992). Stem-water Potential as a Sensitive Indicator of Water Stress in Prune Trees (*Prunus domestica* L. cv. French). J. Am. Soc. Hortic. Sci..

[8] Chalmers D.J., Mitchell P.D., Van Heek L. (1981). Control of Peach Tree Growth and Productivity by Regulated Water Supply, Tree Density, and Summer Pruning. J. Am. Soc. Hortic. Sci..

[9] Chai Q., Gan Y., Zhao C., Xu H.L., Waskom R.M., Niu Y., Siddique K.H. (2016). Regulated deficit irrigation for crop production under drought stress. Agron. Sustain. Dev..

[10] Calderón-Orellana A. (2020). Challenges Associated with a Successful Management of Regulated Deficit Irrigation in Commercial Fresh-Fruit Production. Agric. Res. Technol. Open Access J..

[11] Girona J., Marsal J., Arbones A., Dejong T.M. (2004). A compararison of the combined effect of water stress and crop load on fruit growth during different phenological stages in young peach trees. J. Hortic. Sci. Biotechnol..

[12] Leib B.G., Caspari H.W., Redulla C.A., Andrews P.K., Jabro J.J. (2006). Partial rootzone drying and deficit irrigation of “Fuji” apples in a semi-arid climate. Irrig. Sci..

[13] Hajlaoui H., Maatallah S., Guizani M., Boughattas N.E., Guesmi A., Ennajeh M., Dabbou S., Lopez-Lauri F. (2022). Effect of regulated deficit irrigation on agronomic parameters of three plum cultivars (*Prunus salicina* L.) under semi-arid climate conditions. Plants.

[14] Blanco V., Blaya-Ros P.J., Torres-Sánchez R., Domingo R. (2020). Influence of regulated deficit irrigation and environmental conditions on reproductive response of sweet cherry trees. Plants.

[15] Pérez-Pastor A., Ruiz-Sánchez M.C., Martínez J.A., Nortes P.A., Artes F., Domingo R. (2007). Effect of deficit irrigation on apricot fruit quality at harvest and during storage. J. Sci. Food Agric..

[16] Goldhamer D., Sibbett G., Phene R., Katayama D. (1994). Early irrigation cutoff has little effect on French prune production. Calif. Agric..

[17] Lampinen B.D., Shackel K.A., Southwick S.M., Olson B., Yeager J.T., Goldhamer D. (1995). Sensitivity of Yield and Fruit Quality of French Prune to Water Deprivation at Different Fruit Growth Stages. Am. Soc. Hortic. Sci..

[18] Maldera F., Pietro S., Camposeo S. (2024). Architectural approach to evaluate the design and management of almond cultivars suitable for super high-density orchards. Front. Plant Sci..

[19] Casanova-Gascón J., Figueras-Panillo M., Iglesias-Castellarnau I., Martín-Ramos P. (2019). Comparison of SHD and open-center training systems in almond tree orchards cv. “Soleta”. Agronomy.

[20] Buler Z., Mika A. (2011). Intensive Plum Orchard with Summer Training and Pruning. Adv. Hort. Sci..

[21] Sánchez-Piñero M., Corell M., Moriana A., Pérez-López D., de Sosa L.L., Medina-Zurita N., Castro-Valdecantos P., Martín-Palomo M. (2024). Response of young super-high density table olive orchard (*Manzanilla de Sevilla*) to different water stress levels considering an accurate determination of endocarp development. Agric. Water Manag..

[22] Lordan J., Zazurca L., Maldonado M., Torguet L., Alegre S., Miarnau X. (2019). Horticultural performance of “Marinada” and “Vairo” almond cultivars grown on a genetically diverse set of rootstocks. Sci. Hortic..

[23] Opazo I., Toro G., Salvatierra A., Pastenes C., Pimentel P. (2020). Rootstocks modulate the physiology and growth responses to water deficit and long-term recovery in grafted stone fruit trees. Agric. Water Manag..

[24] Jiménez S., Dridi J., Gutiérrez D., Moret D., Irigoyen J.J., Moreno M.A., Gogorcena Y. (2013). Physiological, biochemical and molecular responses in four Prunus rootstocks submitted to drought stress. Tree Physiol..

[25] Yahmed J.B., Ghrab M., Mimoun M.B. (2016). Eco-physiological evaluation of different scion-rootstock combinations of almond grown in Mediterranean conditions. Fruits.

[26] Opazo I., Toro G., Solis S., Salvatierra A., Franck N., Albornoz F., Pimentel P. (2019). Late reduction on transpiration is an important trait for water deficit tolerance in interspecific Prunus rootstock hybrids. Theor. Exp. Plant Physiol..

[27] Hayat F., Li J., Iqbal S., Khan U., Ali N.A., Peng Y., Hong L., Asghar S., Javed H.U., Li C. (2023). Hormonal interactions underlying rootstock-induced vigor control in horticultural crops. Appl. Sci..

[28] Chen K., Li G.J., Bressan R.A., Song C.P., Zhu J.K., Zhao Y. (2020). Abscisic acid dynamics, signaling, and functions in plants. J. Integr. Plant Biol..

[29] Bambach N.E., Rhoades A.M., Hatchett B.J., Jones A.D., Ullrich P.A., Zarzycki C.M. (2022). Projecting climate change in South America using variable-resolution Community Earth System Model: An application to Chile. Int. J. Climatol..

[30] Gregory P., Atkinson C., Glyn A., Else M., Fernandez-Fernandez F., Harrison R., Schmidt S. (2013). Contributions of roots and rootstocks to sustainable, intensified crop production. J. Exp. Bot..

[31] Milošević A., Marinković J., Branislava T. (2012). Mitigating abiotic stress in crop plants by microorganisms. Matica Srpska J. Nat. Sci..

[32] Bouremani N., Cherif-Silini H., Silini A., Bouket A.C., Luptakova L., Alenezi F.N., Belbahri L. (2023). Plant growth-promoting rhizobacteria (PGPR): A rampart against the adverse effects of drought stress. Water.

[33] Gouda S., Kerry R.G., Das G., Paramithiotis S., Shin H.S., Patra J.K. (2018). Revitalization of plant growth promoting rhizobacteria for sustainable development in agriculture. Microbiol. Res..

[34] Liu F., Ma H., Liu B., Du Z., Ma B., Jing D. (2023). Effects of plant growth-promoting rhizobacteria on the physioecological characteristics and growth of walnut seedlings under drought stress. Agronomy.

[35] Gasic K., Preece J.E. (2014). Register of New Fruit and Nut Cultivars List 47. HortSci. Horts.

[36] Bellvert J., Nieto H., Pelechá A., Jofre-Čekalović C., Zazurca L., Miarnau X. (2021). Remote sensing energy balance model for the assessment of crop evapotranspiration and water status in an almond rootstock collection. Front. Plant Sci..

[37] Iglesias I., Giné-Bordona J., Garanto X., Reig G. (2019). Rootstock affects quality and phytochemical composition of “Big Top” nectarine fruits grown under hot climatic conditions. Sci. Hortic..

[38] Allen R.G., Pereira L.S., Raes D., Smith M. (1998). Crop Evapotranspiration-Guidelines for Computing Crop Water Requirements-FAO Irrigation and Drainage Paper 56.

[39] D’Urso G., Richter K., Calera A., Osann M.A., Escadafal R., Garatuza-Payan J., Hanich L., Perdigão A., Tapia J.B., Vuolo F. (2010). Earth Observation products for operational irrigation management in the context of the PLEIADeS project. Agric. Water Manag..

[40] Balbontín C., Agricultural Satellite Platform to Determine Crop Irrigation (2021). Instituto de Investigaciones Agropecuarias (INIA). https://maps.spiderwebgis.org/login/?custom=plas.

[41] Jovanovic N., Pereira L.S., Paredes P., Pôças I., Cantore V., Todorovic M. (2020). A review of strategies, methods and technologies to reduce non-beneficial consumptive water use on farms considering the FAO56 methods. Agricult. Water Manag..

[42] Reyes-Diaz M., Alberdi M., de la Luz Mora M. (2009). Short-term aluminum stress differentially affects the photochemical efficiency of photosystem II in highbush blueberry genotypes. J. Am. Soc. Hortic. Sci..

[43] Dobereiner J., Baldani V., Baldani J. (1995). Como Isolar e Identificar Bacterias Diazotróficas de Plantas Não-Leguminosas.

[44] Rodriguez E. (1982). Improved medium for isolation of *Azospirillum* spp.. Appl. Environ. Microbiol..

[45] Wells J.M., Bukovac M.J. (1978). Effect of fruit thinning on size and quality of "Stanley" plum (*Prunus domestica* L.). J. Am. Soc. Hortic. Sci..

[46] Basile B., Mariscal M.J., Day K.R., Johnson R.S., DeJong T.M. (2002). Japanese plum (*Prunus salicina* L.) fruit growth: Seasonal pattern of source/sink limitations. J. Am. Pomol. Soc..

[47] Lampinen B.D., Shackel K.A., Southwick S.M., Olson W.H., DeJong T.M. (2004). Leaf and canopy level photosynthetic responses of French prune (*Prunus domestica* L. ‘French’) to stem water potential based deficit irrigation. J. Hortic. Sci. Biotechnol..

[48] Buwalda J.G., Noga G. (1994). Intra-plant differences in leaf chlorophyll fluorescence parameters in perennial fruiting plants. N. Z. J. Crop Hortic. Sci..

[49] Álvarez-Maldini C., Acevedo M., Pinto M. (2021). Hydroscapes: A useful metric for distinguishing iso-/anisohydric behavior in almond cultivars. Plants.

[50] Parent C., Capelli N., Berger A., Crèvecoeur M., Dat J.F. (2008). An overview of plant responses to soil waterlogging. Plant Stress.

[51] Ashraf M.A. (2012). Waterlogging stress in plants: A review. Afr. J. Agric. Res..

[52] Domingo R., Pérez-Pastor A., Ruiz-Sánchez M.C. (2002). Physiological responses of apricot plants grafted on two different rootstocks to flooding conditions. J. Plant Physiol..

[53] Habibi F., Liu T., Shahid M.A., Schaffer B., Sarkhosh A. (2023). Physiological, biochemical, and molecular responses of fruit trees to root zone hypoxia. Environ. Exp. Bot..

[54] Bhusal N., Kim H.S., Han S.G., Yoon T.M. (2020). Photosynthetic traits and plant–water relations of two apple cultivars grown as bi-leader trees under long-term waterlogging conditions. Environ. Exp. Bot..

[55] Shackel K., Prichard T., Schwankl J. (2012). Irrigation Scheduling and Tree Stress. Prune Production Manual.

[56] Meisner A., Jacquiod S., Snoek B.L., Ten Hooven F.C., Van der Putten W.H. (2018). Drought legacy effects on the composition of soil fungal and prokaryote communities. Front. Microbiol..

[57] Pallo Y. (2011). Evaluación de Soportes Sólidos y Líquidos, para la Producción de un Biofertilizante a base de *Azospirillum* spp. Aplicable al Cultivo de Maíz (*Zea mayz* L.). Bachelor’s Thesis.

[58] Cassán F., Coniglio A., López G., Molina R., Nievas S., Le Noir de Carlan C., Donadio F., Torres D., Rosas S., Olivera-Pedrosa F. (2020). Everything you must know about *Azospirillum* and its impact on agriculture and beyond. Biol. Fertil. Soils..

[59] De-Bashan L.E., Holguin G., Glick B.R., Bashan Y. (2007). Plant growth-promoting bacteria for agricultural and environmental purposes. Agricultural Microbiology. Fungi, Bacteria, Micro and Macrofauna, Biological Control, Plant-Microorganism.

[60] Xie J., Dawwam G.E., Sehim A.E., Li X., Wu J., Chen S., Zhang D. (2021). Drought stress triggers shifts in the root microbial community and alters functional categories in the microbial gene pool. Front. Microbiol..

[61] Barnard R.L., Osborne C.A., Firestone M.K. (2013). Responses of soil bacterial and fungal communities to extreme desiccation and rewetting. ISME J..

[62] Manzanera M. (2021). Dealing with water stress and microbial preservation. Environ. Microbiol..

[63] Denef K., Six J., Bossuyt H., Frey S.D., Elliott E.T., Merckx R., Paustian K. (2001). Influence of dry–wet cycles on the interrelationship between aggregate, particulate organic matter, and microbial community dynamics. Soil Biol. Biochem..

[64] González Y. (2010). Actinomycetes: A Vision as Plant Growth Promoters. Bachelor’s Thesis.

